# Association between the CD40 rs1883832 polymorphism and Graves' disease risk: a meta-analysis

**Published:** 2019-01-23

**Authors:** Xiao-Xiong Wang, Xiao-Xia Wang, Tong Chen

**Affiliations:** 1 Department of Ophthalmology, Beijing Hospital, National Center of Gerontology, Beijing, China; 2 Department of Endocrinology, Beijing Hospital, National Center of Gerontology, Beijing, China

**Keywords:** CD40, Graves' disease, meta-analysis, polymorphism

## Abstract

This meta-analysis aims to evaluate whether the *CD40* rs1883832 polymorphism is associated with Graves' disease (GD) risk in different populations. We performed a systematic literature search in China National Knowledge Infrastructure (CNKI), Web of Science, and Pubmed databases to identify case-control association studies on the association between rs1883832 and GD risk. For each study we calculated the odds ratios (OR) and 95 % confidence intervals (CI) assuming dominant, recessive and homozygote models. We then calculated pooled ORs and 95 % CIs. After applying inclusion and exclusion criteria, 17 studies involving 4707 cases and 4215 controls were included in the meta-analysis. The results showed that rs1883832 was associated with GD risk in Asians under dominant (CT + TT vs CC, OR=0.67, 95 % CI: 0.56-0.81, P<0.001), recessive (TT vs CT + CC, OR=0.58, 95 % CI: 0.47-0.72, P<0.001), and homozygote (TT vs CC, OR=0.49, 95 % CI: 0.37-0.64, P<0.001) models. In Caucasians, rs1883832 was associated with GD risk under the dominant model (CT + TT vs CC, OR=0.82, 95 % CI: 0.68-0.99, P=0.042). Besides GD, we evaluated the relation of rs1883832 with Graves' ophthalmopathy (GO), finding that rs1883832 was associated with GO under the dominant model (CT + TT vs CC, OR=0.82, 95 % CI: 0.69-0.98, P=0.031). The findings of our meta-analysis suggest that the *CD40 *rs1883832 polymorphism is protective against GD and GO in Asians and Caucasians.

## Introduction

Graves' disease (GD) is the most common cause of hyperthyroidism, affecting approximately 2 % of women and 0.2 % of men (Smith and Hegedüs, 2016[27]). Thyroid receptor antibodies activate the thyrotropin-releasing hormone receptor of the thyroid hormone producing cell, leading to over-activity of the thyroid gland. Increased serum levels of the thyroid hormone cause clinical symptoms including excessive physical activity, tremor, weight loss, palpitations, and tachycardia (Smith and Hegedüs, 2016[27]). In addition to hyperthyroidism, up to half of GD patients develop the ocular manifestation of the disease, known as Graves' ophthalmopathy (GO) (Shanmuganathan et al., 2015[26]). The pathogenesis of GD remains largely unknown, but it is believed to result from a complex interaction between the immune system, genetic susceptibility genes and environmental factors. There is abundant epidemiologic evidence showing the involvement of several loci in susceptibility to GD, including the human leukocyte antigen (HLA) region on chromosome 6p21, lymphoid protein tyrosine phosphatase (*PTPN22*) on chromosome 1p13, and cytotoxic T lymphocyte antigen 4 (*CTLA-4*) on chromosome 2q33 (Smith and Hegedüs, 2016[27]).

CD40 is a type I transmembrane protein belonging to the tumor necrosis factor (TNF) superfamily. It is expressed on a wide variety of cells including dendritic cells, monocytes, B cells, thyroid follicular cells, and fibroblasts (Clark, 2014[5]; Payuhakrit et al., 2015[24]). The activation of CD40 by CD40 ligand (CD40L) plays a pivotal role in the activation of macrophages and T cell priming. In addition, the CD40-CD40L pathway is involved in the sequence of crucial costimulatory events required for germinal center formation, B-cell proliferation, immunoglobulin class switching, antibody secretion and the rescue of B cells from apoptosis (Clark, 2014[5]). CD40-CD40L interactions can switch the immune response to the Th2 pathway and regulate humoral immunity (Tomer et al., 2002[31]). Clinical studies showed that serum concentrations of soluble forms of CD40L were increased in patients with active GD, and the serum TNF-α:sCD40L ratio was a marker for disease activity (Yamamoto et al., 2012[34]). In animal models of GD, up-regulation of CD4 expression was observed in the thyroid (Ye et al., 2012[36]). Moreover, thyroidal CD40 overexpression augmented the production of thyroid-specific autoantibodies, leading to more severe experimental autoimmune GD (Huber et al., 2012[11]). Therefore, CD40 may play an important role in the pathogenesis of GD. 

Within the human* CD40* gene, a functional C/T polymorphism (rs1883832) in the Kozak sequence of the 5' UTR has received much attention. The major allele of this polymorphism enhances the efficiency of CD40 mRNA translation (Blanco-Kelly et al., 2010[2]). In recent years, efforts have been put into assessing the association of rs1883832 with GD risk, but the findings are inconsistent. The purpose of this study was to conduct a meta-analysis of published data on the association between rs1883832 and GD risk in different ethnic groups.

## Materials and Methods

### Study identification

A computer-based literature search was conducted on China National Knowledge Infrastructure (CNKI), Web of Science, and Pubmed online databases to identify studies on the association of rs1883832 with GD risk. The search strategy included using the keywords “CD40, gene, polymorphism, association, risk, and Graves' disease”. Searches were not limited by date restrictions. All the identified publications were evaluated for relevance by two independent reviewers, on the basis of their titles and abstracts. The full text of selected studies was obtained and evaluated for eligibility. In addition to database search, the reference lists of all included studies and relevant reviews were carefully scrutinized for additional publications. Final eligibility of studies was decided by consensus. 

### Selection criteria

Case-control studies were included if: (1) dealt with the association between the rs1883832 polymorphism and GD risk, (2) provided the raw or summary data necessary to calculate the effect size, and (3) published as full-length articles or letters in peer reviewed journals in Chinese or English. Studies that assessed posttranscriptional factors, such as protein and mRNA expression, rather than genotypic variations were excluded. Studies that used animal populations were excluded.

### Data extraction

Two reviewers independently extracted data from the included studies using pre-defined criteria and compared data to achieve maximum reliability. The following information was obtained from each publication: first author's name, published year, race of the study populations, sample size, genotyping method, source of controls, age of subjects, female proportion, and genotype frequencies. Disagreements were resolved by discussion and consensus. We did not contact study authors for missing or unclear information because it was not a reliable method. All study methodology conformed to the Meta-analysis of Observational Studies in Epidemiology (MOOSE) criteria (Stroup et al., 2000[28]).

### Statistical analyses

All statistical analyses were undertaken using Stata version 12.0 (Stata Corporation, College Station, TX). Odds ratios (OR) with 95 % confidence intervals (CI) were used to assess the size and strength of association between rs1883832 and GD risk. Heterogeneity (true variance of effect size across studies) was assessed using the Cochran Q-test and the I^2^. Large heterogeneity was typically defined as I^2^>75 %.

A P-value of >0.10 for the Cochran Q-test was considered to indicate a lack of heterogeneity across studies. All meta-analyses were performed by using either the DerSimonian-Laird random-effects model or the Mantel-Haenszel fixed-effects model, which depended on the heterogeneity (DerSimonian and Laird, 1986[6]; Mantel and Haenszel, 1959[21]). The Z-test test was used to determine the significance of the combined OR; P<0.05 was considered as statistically significant. A forest plot was used to graphically present the calculated pooled ORs and the 95 % CIs. To test the robustness of our findings, we also conducted a cumulative meta-analysis by date of the eligible studies using a random-effects model. Sensitivity analysis was carried out excluding studies whose allele frequencies in controls exhibited significant deviation from the Hardy-Weinberg equilibrium (HWE). Stratified analyses were conducted according to ethnicity. Besides GD, we evaluated the relation of GO with the rs1883832 polymorphism. To test the deviation from HWE, we used a publicly available program (http://ihg.gsf.de/cgi-bin/hw/hwa1.pl). Meta-regression was undertaken to assess the potential sources of heterogeneity identified in the meta-analysis. Publication bias was assessed visually using a funnel plot and tested with the Begg rank correlation test and the Egger linear regression approach.

## Results

### Characteristics of the studies

**Figure 1:**
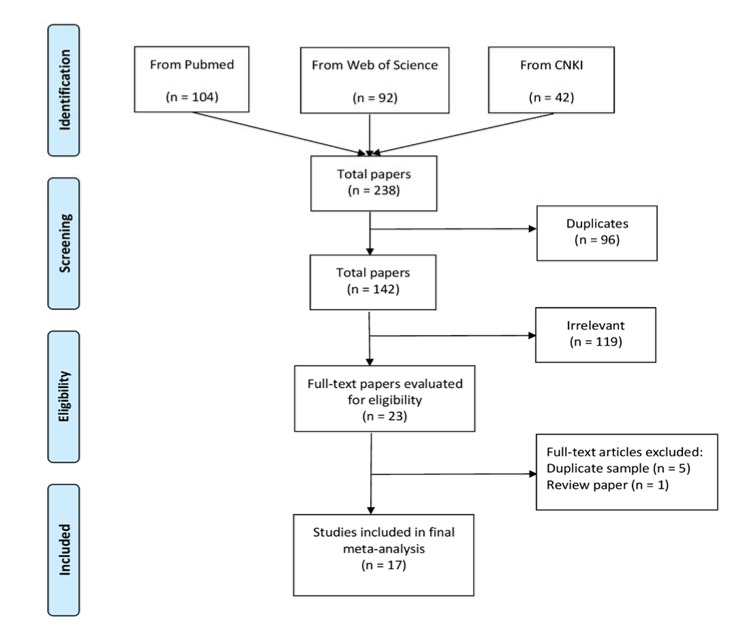
Flow diagram of studies included in the meta-analysis

Details of the search results and study inclusion process are shown in Figure 1(Fig. 1). The literature search identified 238 citations. Of which, 96 duplicates were excluded. One hundred and forty-two publications were then checked for relevance based on the title/abstract screening, from which 119 studies were excluded. Twenty-three full-text studies were carefully read for eligibility. Overall, 17 eligible studies on the association between rs1883832 and GD risk met the inclusion criteria and were included in the meta-analysis (Ban et al., 2006[1]; Chen et al., 2015[4]; Heward et al., 2004[8]; Houston et al., 2004[9]; Hsiao et al., 2008[10]; Inoue et al., 2012[13]; Jacobson et al., 2007[14]; Kim et al., 2003[15]; Kurylowicz et al., 2005[16]; Luo et al., 2006[18]; Ma et al., 2010[19]; Makni et al., 2007[20]; Mukai et al., 2005[22]; Su et al., 2009[29]; Sun et al., 2007[30]; Wang et al., 2017[32]; Yang et al., 2012[35]). The combined population size of the 17 studies totalled included 8922 individuals (4707 GD patients and 4215 controls). The eligible studies were published between 2003 and 2017 and were conducted in a wide range of geographic settings. Among them, 12 studies involving 2614 cases and 2036 controls were performed in Asian populations (Ban et al., 2006[1]; Chen et al., 2015[4]; Hsiao et al., 2008[10]; Inoue et al., 2012[13]; Kim et al., 2003[15]; Luo et al., 2006[18]; Ma et al., 2010[19]; Mukai et al., 2005[22]; Su et al., 2009[29]; Sun et al., 2007[30]; Wang et al., 2017[32]; Yang et al., 2012[35]), while five studies including 2093 cases and 2179 controls were undertaken in Caucasians (Heward et al., 2004[8]; Houston et al., 2004[9]; Jacobson et al., 2007[14]; Kurylowicz et al., 2005[16]; Makni et al., 2007[20]). Characteristics of the included studies are listed numerically and summarized in Table 1(Tab. 1) (References in Table 1: Ban, 2006[1]; Chen, 2015[4]; Heward, 2004[8]; Houston, 2004[9]; Hsiao, 2008[10]; Inoue, 2012[13]; Jacobson, 2007[14]; Kim, 2003[15]; Kurylowicz, 2005[16]; Luo, 2006[18]; Ma, 2010[19]; Makni, 2007[20]; Mukai, 2005[22]; Su, 2009[29]; Sun, 2007[30]; Wang, 2017[32]; Yang, 2012[35]).

### Data analysis

**Figure 2:**
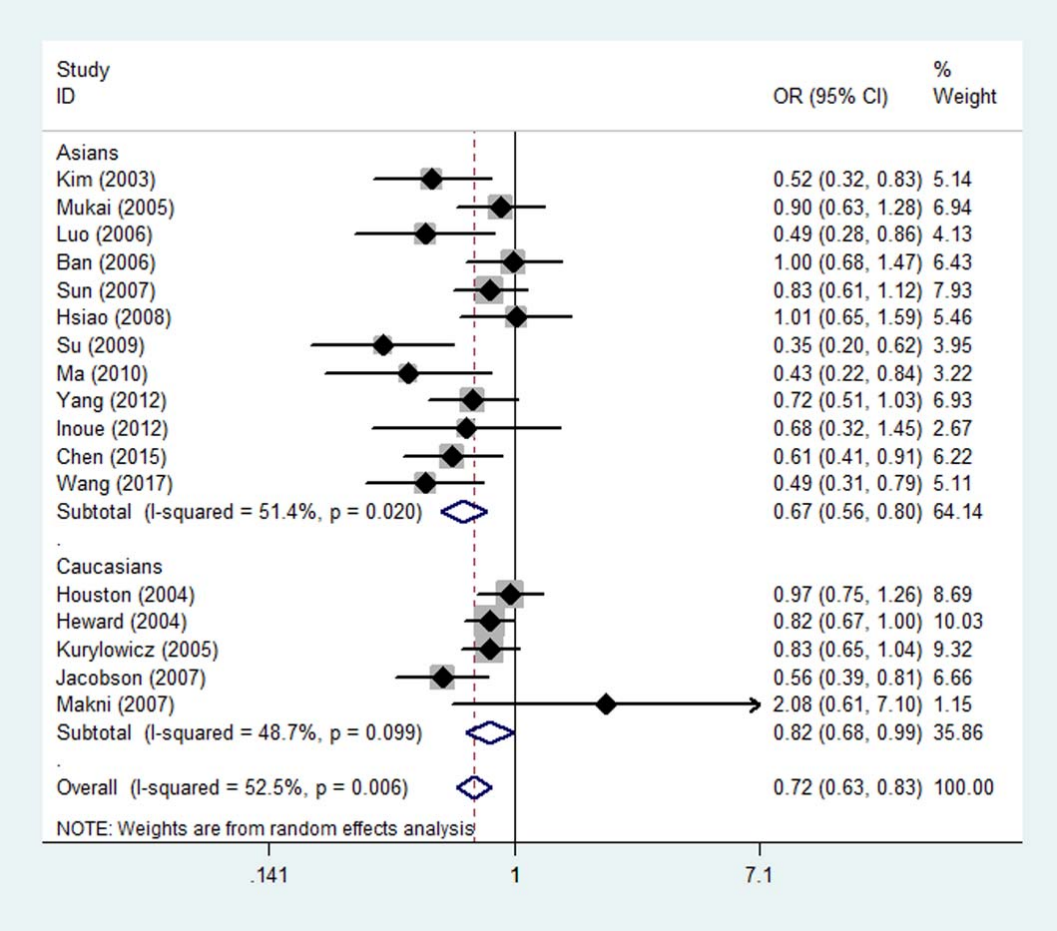
Forest plot showing the association between the *CD40* rs1883832 polymorphism and Graves' disease risk assuming a dominant (TT + CT vs. CC) model

**Figure 3:**
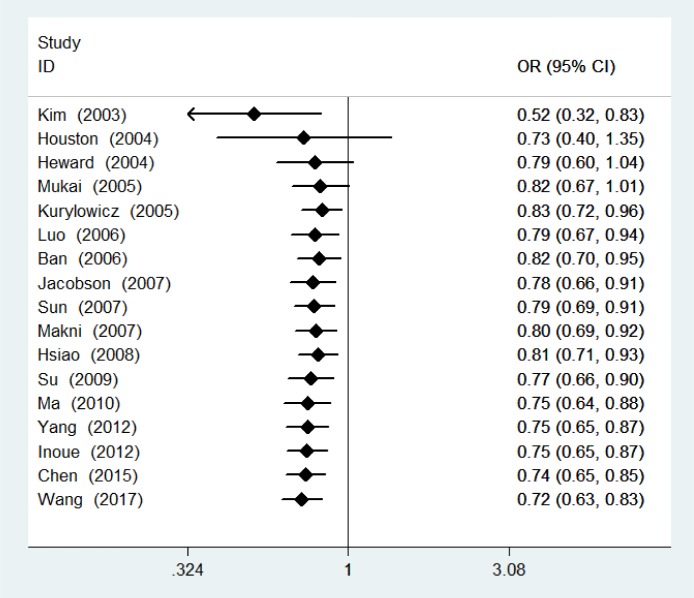
Cumulative meta-analysis for the association between the *CD40* rs1883832 polymorphism and Graves' disease risk assuming a dominant (TT + CT vs. CC) model

Meta-analysis of genotype data for dominant, recessive, and homozygote models is shown in Table 2(Tab. 2) and Figure 2(Fig. 2). Pooling data from all eligible studies supported an association between the *CD40* rs1883832 polymorphism and GD risk under dominant (CT + TT vs CC, OR=0.72, 95 % CI: 0.63-0.83, P<0.001), recessive (TT vs CT + CC, OR=0.60, 95 % CI: 0.50-0.73, P<0.001), and homozygote (TT vs CC, OR=0.56, 95 % CI: 0.43-0.71, P<0.001) models. Similar results were found for allele frequency (T allele vs C allele, OR=0.75, 95 % CI: 0.67-0.84, P<0.001). Stratified analyses according to ethnicity showed that the rs1883832 polymorphism was associated with GD risk in Asians under dominant (CT + TT vs CC, OR=0.67, 95 % CI: 0.56-0.81, P<0.001), recessive (TT vs CT + CC, OR=0.58, 95 % CI: 0.47-0.72, P<0.001), and homozygote (TT vs CC, OR=0.49, 95 % CI: 0.37-0.64, P<0.001) models. In Caucasians, there was an association of this polymorphism with GD under the dominant model (CT + TT vs CC, OR=0.82, 95 % CI: 0.68-0.99, P=0.042). Besides GD, we evaluated the relation of rs1883832 with GO, finding that rs1883832 was associated with GO under the dominant model (CT + TT vs CC, OR=0.82, 95 % CI: 0.69-0.98, P=0.031). Sensitivity analysis was performed excluding studies whose controls significantly departed from HWE (Luo et al., 2006[18]; Ma et al., 2010[19]); the results remained practically unchanged (pooled OR=0.75, 95 % CI: 0.66-0.86, P<0.001 for the dominant model; pooled OR=0.66, 95 % CI: 0.57-0.76, P<0.001 for the recessive model; pooled OR=0.61, 95 % CI: 0.48-0.77, P<0.001 for the homozygote model) (Table 2(Tab. 2)). The cumulative meta-analysis showed a significant and stable association between rs1883832 and GD risk under the dominant model (CT + TT vs CC) over time and the trend of the effect estimate stabilized by 2010 (Figure 3(Fig. 3); References in Figure 3: Ban, 2006[1]; Chen, 2015[4]; Heward, 2004[8]; Houston, 2004[9]; Hsiao, 2008[10]; Inoue, 2012[13]; Jacobson, 2007[14]; Kim, 2003[15]; Kurylowicz, 2005[16]; Luo, 2006[18]; Ma, 2010[19]; Makni, 2007[20]; Mukai, 2005[22]; Su, 2009[29]; Sun, 2007[30]; Wang, 2017[32]; Yang, 2012[35]). Significant between-study heterogeneity was identified (Cochran Q-test, P<0.10; I^2^ ranging from 31.2 to 63.3 %) (Table 2(Tab. 2)). Meta-regression showed that sample size (P=0.001) and publication year (P=0.003) were the major factors affecting between-study heterogeneity.

### Publication bias

**Figure 4:**
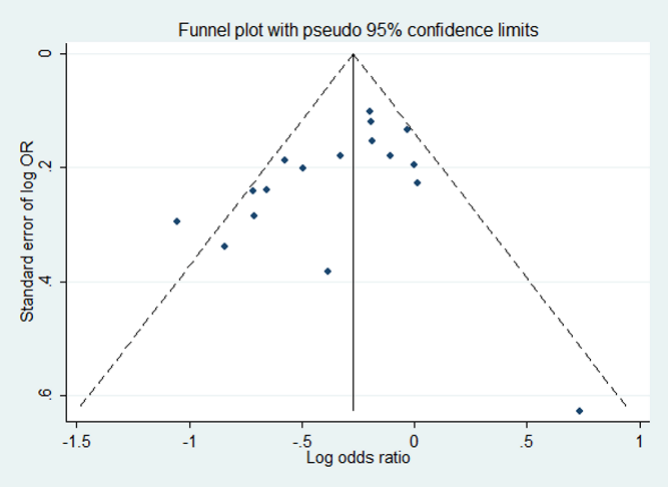
Funnel plot for publication bias amongst the studies used to obtain pooled odds ratio of rs1883832 under the dominant model

Visual inspection of a funnel plot for asymmetry revealed no obvious indication of publication bias (Figure 4(Fig. 4)). In addition, the results of Egger's test and Begg's test suggested no evidence for publication bias (P=0.104 and P=0.108, respectively). 

## Discussion

To our knowledge, this is the largest and most comprehensive meta-analysis examining the relationship between the *CD40* rs1883832 polymorphism and risk of GD. The results suggested that rs1883832 was protective against GD in both Asians and Caucasians. In addition, our meta-analysis identified a negative association between rs1883832 and GO. 

Several previous meta-analyses were performed on the topic. The meta-analysis by Kurylowicz et al. (2005[16]) evaluated the association between rs1883832 and GD risk using five case-control studies, whereas Makni et al. (2007[20]) included seven studies to assess the association. Due to small sample sizes, neither of the two meta-analyses performed subgroup analysis. In addition, these meta-analyses did not have sufficient statistical power to derive a reliable conclusion. Recently, Li and colleagues performed a meta-analysis to investigate the relationship between rs1883832 and GD risk among 4214 cases and 3851 controls in 14 studies (Li et al., 2012[17]). Although Li et al. (2012[17]) found an association between rs1883832 and GD risk, they did not further evaluate race-specific effects and the association in each ethnic group remained unclear. Significant between-study heterogeneity was identified in the Li et al. meta-analysis (Cochran Q-test, P=0.004; I^2^=57.6 %), but the potential sources of heterogeneity were not evaluated. It was noteworthy that none of the above-mentioned meta-analyses assessed the relationship between rs1883832 and GO, a condition associated with GD that primarily affected the extraocular muscles. In this meta-analysis, we evaluated the association between rs1883832 and GD risk with a total of 4707 GD patients and 4215 controls, having the largest sample size among all meta-analyses on the topic. Besides overall analyses, we conducted subgroup analyses by ethnicity and found a negative association between rs1883832 and GD risk in Asians and Caucasians, respectively. Our study further added to the literature by identifying an association of GO with rs1883832. Concerning the heterogeneity among the included studies, we performed a meta-regression analysis and found publication year and sample size as major contributors to heterogeneity. 

The *CD40* gene is a member of the TNF receptor family, expressed on the surface of a variety of cells, including B cells, macrophages and thyroid follicular cells. CD40 plays an important role in effector T cell development, B cell differentiation, immunoglobulin production, and isotype switching (Clark, 2014[5]). Aberrant CD40 expression have been implicated in GD susceptibility. Animal studies showed significant elevation of CD40 expression in the thyroid of experimental GD (Ye et al., 2012[36]). *In vivo* blockade of CD40 significantly suppressed murine experimental autoimmune thyroiditis while transgenic mouse models constitutively overexpressing thyroidal CD40 augmented the production of thyroid-specific antibodies, resulting in more severe experimental autoimmune GD (Carayanniotis et al., 1997[3]). It is known that the minor allele of rs1883832 decreases the efficiency of CD40 mRNA translation and associates with reduced CD40 expression (Blanco-Kelly et al., 2010[2]). Given the pivotal role of CD40 in the development of GD and autoimmunity, reduced CD40 expression induced by rs1883832 may decrease the risk of developing GD. Our meta-analysis found differences between Asians and Caucasians regarding the association between rs1883832 and GD risk, suggesting that rs1883832 may have different effects on GD risk according to genetic background.

GO is the most frequently occurring extra-thyroidal manifestation GD, affecting 20 % to 50 % of GD patients (Shanmuganathan et al., 2015[26]). Clinical studies demonstrated that orbital fibroblasts and peripheral blood fibrocytes from patients with GO expressed substantially higher levels of CD40 (Douglas et al., 2014[7]; Hwang et al., 2009[12]; Pawlowski et al., 2015[23]). These cells were activated through CD40 and contributed to the development of GO by producing inflammatory cytokines, promoting the recruitment of inflammatory cells, and regulating tissue reactivity and remodeling (Douglas et al., 2014[7]; Sempowski et al., 1998[25]; Wu et al., 2016[33]). Our meta-analysis found an association between rs1883832 and GO, which was in line with previous clinical studies and supported an important role of CD40 in the development of GO. 

Some limitations of our study need to be considered. Firstly, the eligible studies in our meta-analysis were mainly performed on Asian and Caucasian subjects. Data from other ethnic groups were unavailable. Future association studies should be conducted in other ethnic groups including Latin Americans and Africans, which would yield a more global and profound picture of the role mediated by rs1883832 in GD pathogenesis. Secondly, the association between rs1883832 and clinical features of GD including age of onset and thyroid autoantibodies were not taken into account in our meta-analysis owing to a lack of published data. It would be valuable to evaluate the relation of rs1883832 with GD features, which could clarify the role of rs1883832 in different subsets of GD patients. Thirdly, due to limitations of the data, gene-gene and gene-environment interactions were not analyzed in the meta-analysis. Future studies evaluating the interactions of rs1883832 with environmental factors and HLA polymorphisms will further expand our knowledge of the underlying genetic mechanisms of GD.

In conclusion, the available evidence from the present meta-analysis involving 4707 GD patients and 4215 controls suggests a protective effect of the *CD40* rs1883832 polymorphism on GD in both Asians and Caucasians. Our study also demonstrated a negative association of rs1883832 with GO. Further investigations are warranted to clarify the role of rs1883832 in clinical features of GD and to evaluate gene-gene and gene environment interactions. 

## Conflict of interest

The authors declare that they have no conflict of interest.

## Figures and Tables

**Table 1:**
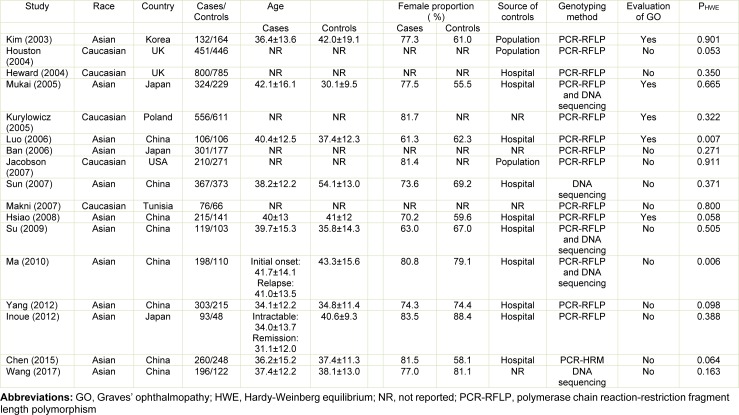
Characteristics of the studies considered in the meta-analysis

**Table 2:**
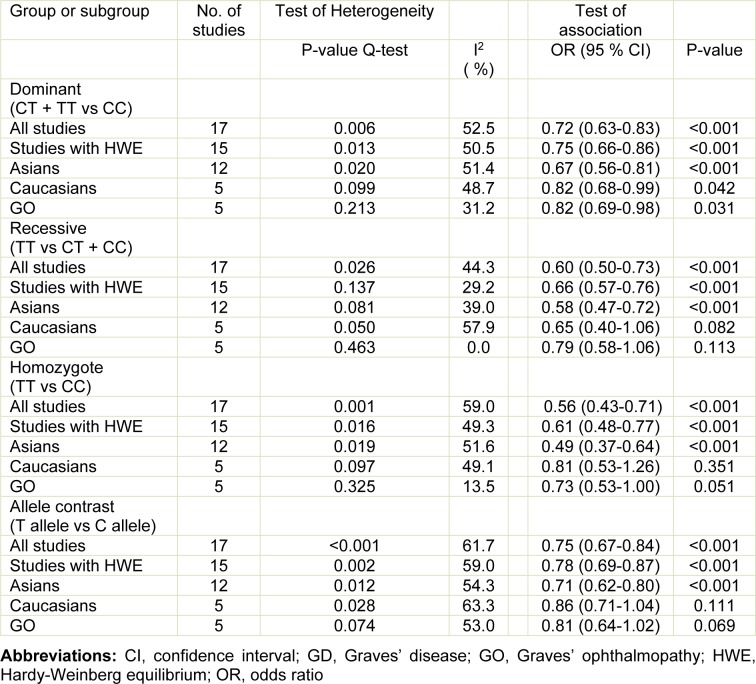
Summary ORs of the meta-analysis on associations of the *CD40* rs1883832 polymorphism with GD
